# A Stability- and Aggregation-Based Method for Heart Rate Estimation Using Photoplethysmographic Signals During Physical Activity

**DOI:** 10.3390/s25144315

**Published:** 2025-07-10

**Authors:** Sabrina C. Crepaldi, Jiabin Wang, Fumiya Matsumoto, Hiroki Takeuchi, Tatsuhiko Watanabe, Yoshiharu Yamamoto

**Affiliations:** 1 SOXAI Inc., Kanagawa 231-0032, Japan; 2 Educational Physiology Laboratory, Graduate School of Education, The University of Tokyo, Tokyo 113-0033, Japanyamamoto@p.u-tokyo.ac.jp (Y.Y.)

**Keywords:** heart rate, PPG, smart ring, active heart rate, dataset

## Abstract

In recent years, the use of photoplethysmography (PPG)-based heart rate detection has gained considerable attention as a cost-effective alternative to conventional electrocardiography (ECG) for applications in healthcare and fitness tracking. Although deep learning methods have shown promise in heart rate estimation and motion artifact removal from PPG signals recorded during physical activity, their computational requirements and need for extensive training data make them less practical for real-world conditions when ground truth data is unavailable for calibration. This study presents a one-size-fits-all approach for heart rate estimation during physical activity that employs aggregation-based techniques to track heart rate and minimize the effects of motion artifacts, without relying on complex machine learning or deep learning techniques. We evaluate our method on four publicly available datasets—*PPG-DaLiA*, *WESAD*, *IEEE_Training*, and *IEEE_Test*, all recorded using wrist-worn devices—along with a new dataset, *UTOKYO*, which includes PPG and accelerometer data collected from a smart ring. The proposed method outperforms the CNN ensemble model for the *PPG-DaLiA* dataset and the *IEEE_Test* dataset and reduces the mean absolute error (MAE) by 1.45 bpm and 5.71 bpm, respectively, demonstrating that effective signal processing techniques can match the performance of more complex deep learning models without requiring extensive computational resources or dataset-specific tuning.

## 1. Introduction

In recent years, there has been a rapid increase in the use of smart wearable devices, such as smartwatches and smart rings, which allow individuals to monitor various physiological parameters in real time [1,2,3]. Namely, the implementation of photoplethysmography (PPG) over conventional electrocardiography (ECG) has gained considerable attention due to its non-invasive and cost-effective nature. Whereas an ECG device measures the heart’s electrical activity through electrodes placed on the body, a PPG device typically uses a light-emitting diode (LED) to shine light into the skin while a photodetector measures the amount of light that is either reflected or transmitted through the tissue. Due to the pulsatile nature of the circulatory system, changes in blood volume are reflected in the absorbed light, which can be used to derive health metrics, including heart rate (HR) [4,5].

In practice, PPG signals are often collected from sensors placed on the wrist or fingers, which can lead to motion artifacts (MAs), especially during physical activity. MAs can compromise the reliability of the measured PPG signal, leading to inaccuracies in heart rate detection and other derived metrics [6,7]. An accurate measurement of heart rate during exercise is especially useful as it provides valuable insights into intensity, recovery, and training effectiveness [8,9]. Beyond fitness monitoring, accurate heart rate measurement during physical movement has applications in clinical contexts, where it can assist in the monitoring of patients with cardiovascular conditions during daily activities [10,11,12].

Despite the growing interest in PPG-based monitoring, the development of algorithms to process PPG signals and mitigate the effects of motion artifacts remains challenging. Spectrum-based approaches form the basis of many of these algorithms, leveraging time-frequency spectra derived from both PPG and acceleration signals to identify and remove periodic components caused by motion artifacts and isolate the frequencies associated with the heart rate [13]. Notable algorithms include *SpaMa* [14] and its enhanced version, *SpaMaPlus* [15], which rely on spectral analysis and focus on identifying and removing peaks in the acceleration spectrum from the PPG spectrum to mitigate motion artifacts. The highest remaining peak in the PPG spectrum is then used to calculate the heart rate. *SpaMaPlus* improves upon *SpaMa* by incorporating a mean filter over recent heart rate estimates and implementing a heart rate tracking step and a reset mechanism to handle abrupt changes in heart rate. More advanced algorithms include TROIKA [16] and JOSS [17], which leverage sparsity-based spectrum estimation and spectral peak tracking techniques to estimate heart rate during intense physical activity, such as treadmill running. TROIKA employs singular spectrum analysis (SSA) for signal decomposition and reconstruction, discarding the SSA components whose frequencies closely match those of motion artifacts detected from accelerometer signals. JOSS extends this approach by formulating a joint sparse signal recovery model, enabling improved spectrum estimation using both PPG and accelerometer data. The spectral peak tracking mechanism reinforces the heart rate estimation by assuming that the spectral peak corresponding to the heart rate remains constant or shifts minimally between overlapping time windows.

Many existing algorithms rely on subject-specific tuning, where several adjustable parameters are tuned to each session specifically, which limits their generalization to daily life where ground truth data are unavailable for calibration [18]. Moreover, these algorithms are often evaluated on short-duration datasets, which do not account for the variability in and complexity of longer-term recordings. The use of longer datasets is critical for ensuring real-world application and robust performance across diverse scenarios. In particular, many landmark studies have relied heavily on the IEEE Signal Processing Cup in 2015 datasets [16,17], which were collected in controlled laboratory settings for a short duration with limited types of physical activities.

More recently, deep learning methods have been proposed as powerful alternatives to classical methods of signal processing and PPG-based heart rate estimation [15,18,19,20]. Several studies have demonstrated that deep learning models, such as convolutional neural networks (CNNs) [21] and recurrent neural networks (RNNs) [22], can outperform traditional algorithms in mitigating motion artifacts and providing accurate heart rate measurements. Biswas et al. combined CNN and LSTM architectures to estimate heart rate and perform biometric identification using pre-processed PPG signals [20]. Similarly, Shen et al. [23] and Shashikumar et al. [24] employed a 50-layer ResNeXt CNN and a wavelet transform followed by a CNN, respectively, to detect atrial fibrillation from PPG signals. Reiss et al. [18] introduced an end-to-end deep learning framework for heart rate estimation, leveraging convolutional neural networks to process the time-frequency spectra of synchronized PPG and accelerometer signals, which significantly outperformed classical approaches.

Unfortunately, deep learning approaches often come with significant computational costs, requiring high processing power and memory resources. Such requirements pose challenges for their integration into smart wearable devices, which are constrained by limited computational capabilities and battery life [25,26].

In this work, we demonstrate that it is possible to achieve performance comparable to state-of-the-art deep learning models without relying on machine learning. Our proposed method highlights that, in certain applications, effective signal processing and algorithmic innovations can bridge the gap traditionally addressed by deep learning. This is particularly relevant for scenarios where machine learning may not be feasible due to computational constraints, limited access to annotated training datasets, or preferences for simpler, interpretable solutions. By advancing non-machine learning approaches, we provide a valuable alternative that expands the toolbox of techniques available for wearable applications, supporting the development of lightweight and scalable solutions.

## 2. Materials and Methods

### 2.1. Datasets

Reiss et al. [18] provide a comprehensive review of the most widely used PPG datasets for HR detection, highlighting their characteristics and summarizing the performance of the common algorithms evaluated on them. In this paper, we evaluate our novel algorithm on these publicly available datasets in addition to a new dataset, *UTOKYO*, which includes 68 PPG and accelerometer recordings of activity sessions collected using a smart ring.

All the publicly available datasets evaluated in this study were recorded using a wrist-worn PPG device and an ECG device for the ground truth. To match the methods used in [18] and to maintain uniformity across all the datasets, the ground truth for the heart rate is calculated over an 8 s sliding window, with a window shift of 2 s. Likewise, the PPG- and accelerometer-derived heart rates are calculated with the same sliding window size and window shift. The choice of the window duration is also supported by the fact that the heart rate does not change instantaneously.

The details of the datasets are outlined in the following paragraphs.

1. *PPG-DaLiA*: This dataset includes a single-channel PPG signal sampled at 64 Hz and three-axis accelerometer data sampled at 32 Hz. To maintain uniformity in the analysis, all the signals were sampled to 32 Hz. The dataset comprises 36 h of data for 15 subjects during a progression of daily activities, which includes sitting, climbing stairs, table soccer, cycling, driving, having lunch, walking, and working. More details on this dataset are provided in [18].
2. *WESAD*: This dataset includes a single-channel PPG signal sampled at 64 Hz and three-axis accelerometer data sampled at 32 Hz. For uniformity, all the signals were sampled to 32 Hz. The dataset comprises data for 15 participants, recorded for approximately 100 min each, and includes a mix of activities, such as reading, watching videos, preparing for and giving a speech, and meditation. More details on this dataset are provided in [27].
3. *IEEE_Training*: This dataset was created for the IEEE Signal Processing Cup in 2015. It includes two-channel PPG signals and three-axis accelerometer data, all sampled at a rate of 125 Hz. Down-sampling of the signals to 25 Hz was performed to match the sampling rate of the original paper [17]. The dataset captures 12 treadmill sessions of approximately 5 min each, performed at varying speeds by different subjects. More details on this dataset are provided in [16,17,18].
4. *IEEE_Test*: This dataset was also developed for the IEEE Signal Processing Cup in 2015, using the same recording parameters and ground truth conditions as *IEEE_Training*. However, for this dataset, 8 different subjects each performed 10 sessions of arm activities, which included 4 sessions of mixed arm exercises and 6 sessions of intensive arm exercises, each lasting approximately 5 min. More details on this dataset are provided in [16,17,18].
5. *UTOKYO*: This dataset includes 7 warm-up sessions, 14 walking sessions, and 47 running sessions, of which 35 were performed outdoors and 12 were performed on an indoor treadmill. The walking and running sessions lasted between 5 and 10 min, whereas the warm-up sessions lasted between 10 and 15 min. The data was collected for 20 different subjects, and each subject collected at least 2 sessions of data. The PPG data was recorded with a sampling rate of 182 Hz, while the three-axis acceleration data was sampled at a rate of 52 Hz. For uniformity, the signals were resampled to 39 Hz. The ground truth for the heart rate was obtained from a chest-worn ECG device as the mean heart rate calculated over 8 s sliding windows, with a window shift of 2 s. However, when deriving the heart rates from the PPG and acceleration signals, a sliding window of 16 s (with the same window shift size) was used compared to the other datasets to avoid having empty windows after outlier removal, as the signals of this dataset contained more noise. During the analysis, the start times of the smart ring and ECG recordings were aligned to ensure synchronization.

Table 1 summarizes the mean and standard deviation of the ECG-derived reference heart rates across all the datasets, with the *UTOKYO* dataset further specified by activity type.

### 2.2. Methodology

#### 2.2.1. Data Collection for the *UTOKYO* Dataset

The dataset was collected from 20 participants of Japanese ethnicity between 20 and 30 years of age, all of whom engaged in regular exercise at the time of the data collection. The activities included running, walking, and warm-up exercises. The PPG data was collected using a smart ring (SOXAI RING 1) worn on the index or middle finger of the dominant hand, depending on participant comfort. The participants were fitted with appropriately sized rings, and the device’s position was adjusted at the start of every session to improve signal acquisition. The smart ring recorded two-channel PPG signals using red (655 nm) and infrared (940 nm) light sources. The data was transmitted to a smartphone in real time via Bluetooth Low Energy (BLE) and saved to a smartphone’s storage every 30 s. Recordings shorter than 30 s were excluded from the analysis. The reference HR data was collected at a rate of 1 Hz using a chest-worn Polar H10 N ECG device from Polar Inc.

#### 2.2.2. Proposed Algorithm

The following algorithm was applied uniformly to both the publicly available datasets and the *UTOKYO* dataset, without any subject-specific or dataset-specific parameter tuning. It should be noted that a better performance can be achieved with the presented method by applying dataset-specific tuning, but the parameters described in this section are presented as a one-size-fits-all solution when calibration is not available.

**Figure 1:**
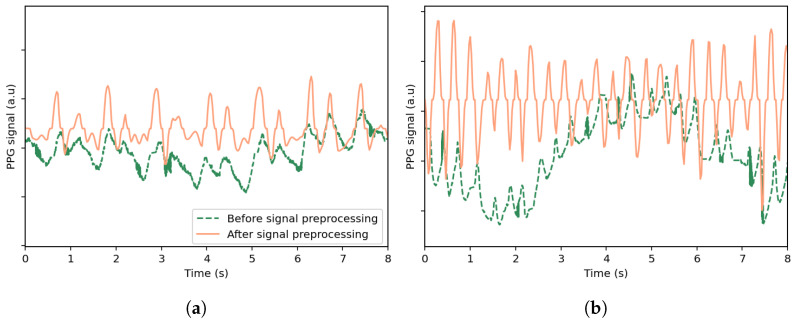
Comparison of the original PPG signal and the signal after preprocessing, shown for selected time windows from the *UTOKYO* dataset for (**a**) a walking session and (**b**) a running session. The preprocessing steps include outlier removal with linear interpolation, bandpass filtering, resampling, and smoothing.

**Figure 2:**
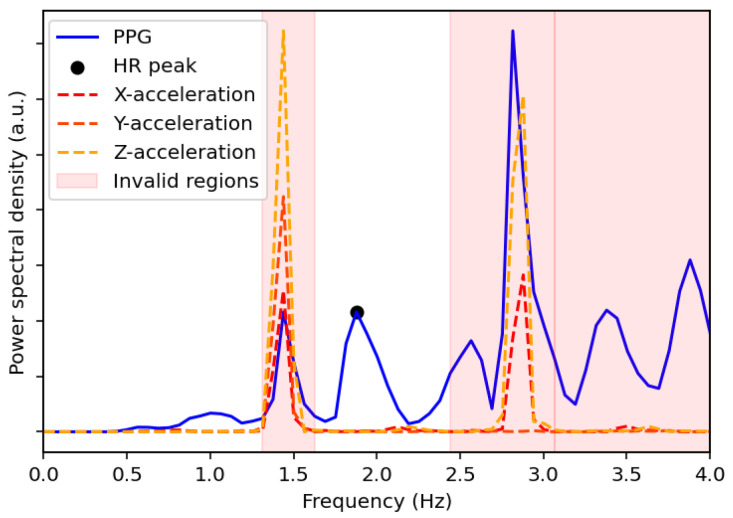
Power spectral density comparison between the PPG signal and the x-, y-, and z-acceleration signals for a selected time window of the *UTOKYO* dataset. The estimated HR peak from the spectral analysis is also labeled, which corresponds to 113 bpm and matches the ground truth measurement. The regions with invalid peaks are also shaded in red.

**Figure 3:**
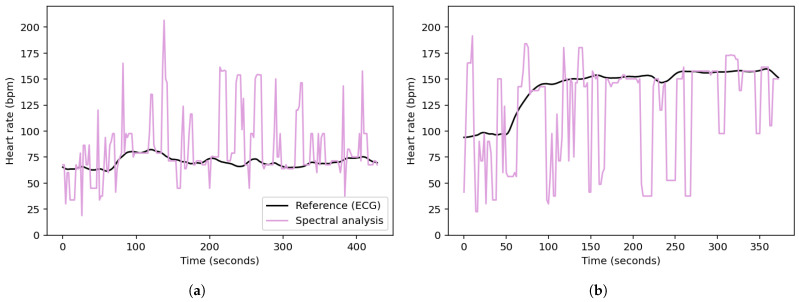
Heart rates obtained from the spectral analysis of (**a**) a walking session and (**b**) a running session from the *UTOKYO* dataset.

**Figure 4:**
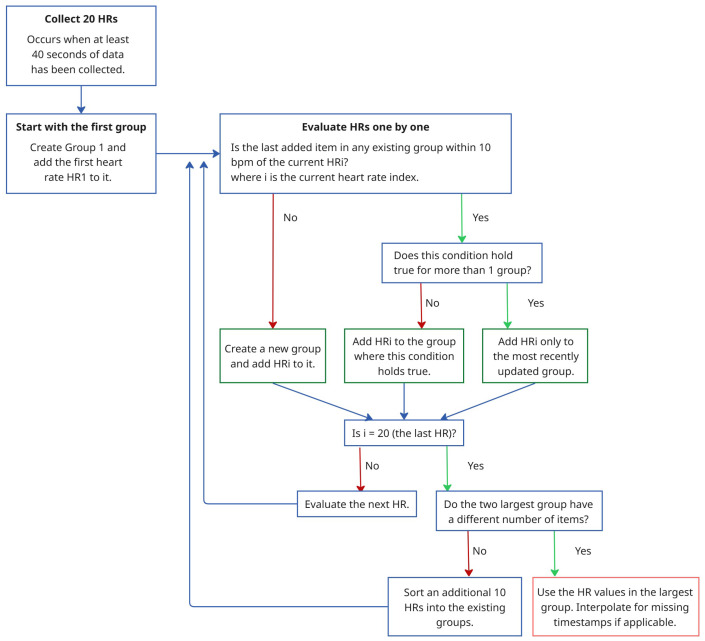
Flowchart illustrating the SAB-HRT procedure.

**Figure 5:**
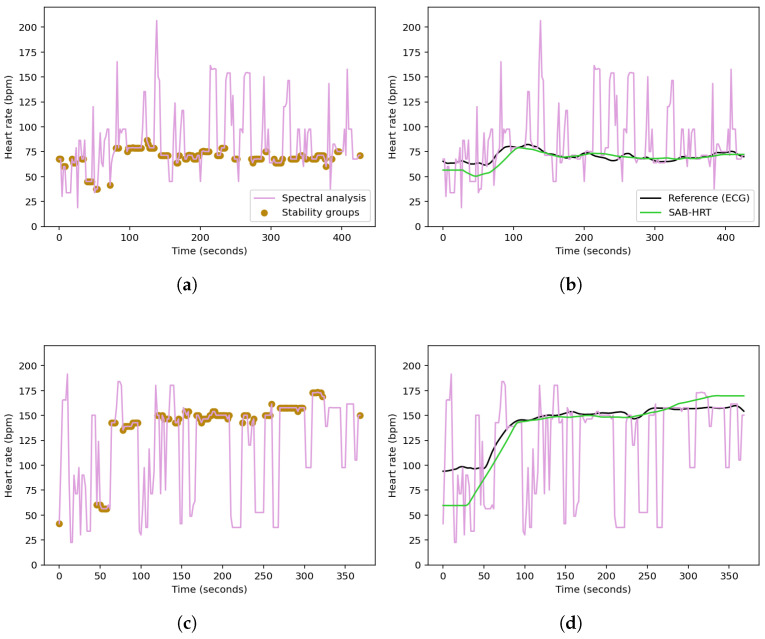
Identification of the stability group points from the spectral heart rate estimates for a specified (**a**) walking session and (**c**) running session from the *UTOKYO* dataset. The post-processing step, where the selected points are connected and smoothed to produce a continuous heart rate signal, are shown in (**b**) and (**d**), respectively.

A. *Signal Preprocessing*
  Outliers in the PPG signal are identified as values that are either negative or exceed three standard deviations above the mean of the positive values in the signal. As such, a value *p[n]* in the PPG signal is considered an outlier if
  (1) $$p\left[n\right]>\mu_{+}+3\sigma_{+}\;\mathrm{or}\;p\left[n\right]<0$$
  where $\mu_{+}$ and $\sigma_{+}$ are the mean and standard deviation computed over the positive values of a specified PPG signal.
  Outlier values are subsequently replaced using linear interpolation between neighboring valid values to ensure continuity. A bandpass filter is then applied to the signal using a fifth-order Butterworth filter to retain frequencies between 0.5 Hz and 5 Hz. Resampling is also performed using cubic interpolation to ensure that the PPG and accelerometer signals have the same sampling frequency. Each signal is first mapped onto a uniformly spaced time axis derived from its original timestamps and then interpolated onto a new uniform time grid at the desired sampling rate. For uniformity, the signals are analyzed using a sliding window and a window shift whose sizes match the analysis of the publicly available datasets. A median filter with a kernel size of 7 and a moving average filter with a window size of 3 are also applied to reduce noise and smooth the signal.
  These operations are defined in the following equations:
  (2) $$\begin{array}{cc} p_{\mathrm{med}}\left[n\right] & =\mathrm{median}\left(p[n-3],\ldots ,p[n+3]\right) \end{array}$$
  (3) $$\begin{array}{cc} p_{\mathrm{average}}\left[n\right] & =\frac{1}{3}\sum_{k=-1}^{1}p_{\mathrm{med}}\left[n+k\right] \end{array}$$
  where $p_{\mathrm{med}}\left[n\right]$ is the output of the median filter, and $p_{\mathrm{average}}\left[n\right]$ is the output of the moving average filter.
  Figure 1 illustrates the effects of preprocessing on selected PPG signal segments from the *UTOKYO* dataset for both walking and running conditions. As shown, the processed signals exhibit clearer pulse peaks and reduced baseline drift.
B. *Spectral Analysis*
  The power spectral density (PSD) and its corresponding frequencies are computed using Welch’s method for the three directions of the acceleration signals and the PPG signal. The PSD peaks are subsequently identified for each signal. Only the frequencies of the two highest PSD peaks from each acceleration direction are retained, resulting in a maximum of six candidate frequencies, which can be combined to form a full set F:
  (4) $$F=\left\{f_{1,x},f_{2,x},f_{1,y},f_{2,y},f_{1,z},f_{2,z}\right\}$$
  where $f_{1,i}$ and $f_{2,i}$ denote the frequencies at the first and second highest PSD peaks of the acceleration signal in the direction i∈{x,y,z}. To avoid redundancy, duplicate frequencies are removed.
  The frequencies of the PPG signal within a defined range of ±0.1 Hz around each unique peak frequency in F are flagged for removal. The removal range is further extended to include neighboring frequencies if their PSD values are within 5% of the PSD of the removed PPG peak. Additionally, all the frequencies beyond a threshold, defined as 5% greater than the highest peak frequency in F, are also removed. The heart rate, *HR*, for the given window is then determined from the frequency, $f_{\mathrm{peak}}$, corresponding to the highest valid peak remaining in the PPG spectrum using the following equation:
  (5) $$HR=60\times f_{\mathrm{peak}}$$
  Figure 2 shows the PSDs of the PPG signal and acceleration signals as a function of frequency for a specified time window of the *UTOKYO* dataset. The second highest peak in the spectrum of the z-axis acceleration aligns with the strongest peak in the PPG spectrum, suggesting that this dominant PPG component is a possible motion artifact. This is confirmed by the reference heart rate for the specified time window, which coincides with the highest valid PPG peak located at around 1.88 Hz (113 bpm).
  Examples of the spectral analysis step applied to specified walking and running sessions from the *UTOKYO* dataset are shown in panels (a) and (b) of Figure 3, respectively.
C. *Stability- and Aggregation-Based Heart Rate Tracking (SAB-HRT)* To ensure accurate heart rate estimation, a heart rate tracking step is implemented. Like many existing algorithms, this step aims to prevent large fluctuations in heart rate between subsequent measurements in a sequence. This step can be applied continuously and begins when 20 HR values are collected, which occurs every 40 s when using a sliding window with a 2 s shift. The algorithm traverses these HR values sequentially to form stability groups, starting by assigning the first HR value to an initial group. Then, for each subsequent HR, the algorithm verifies whether the difference between the last element of each group is within 10 bpm of the current HR. If the difference is within this threshold for one or more groups, the HR is added to the most recently updated group only. Otherwise, a new group is created to accommodate the HR value. Thus, each HR value should be assigned to a single group only. After all the HR values are grouped, the largest group is selected as representing the HR values for this time period. At timestamps with missing HR values within the selected group, the HR values are interpolated to ensure continuity. If multiple groups have the same size, the algorithm extends the analysis window by half its original size (20 s) and repeats the grouping process. The logic for this process is visualized in the flowchart in Figure 4. As a final step, a continuous moving average filter with a 30 s window is applied to smooth the output signal and enhance signal readability. Importantly, the 30 s window is a conservative choice; our tests show that the size can be reduced to 10 s while keeping the MAE difference minimal.
  Panels (a) and (c) in Figure 5 display the stability group points formed by the SAB-HRT method that were identified from the heart rate estimates obtained via the spectral analysis for the specified walking and running sessions, respectively, shown in Figure 3. Figure 5b,d illustrate the process of connecting and smoothing the selected points to address abrupt transitions.

## 3. Results and Discussion

The results are presented as the mean absolute error (MAE) in beats per minute (bpm) for the current method without any subject-specific or dataset-specific tuning, with the same parameters applied uniformly across all the datasets. The MAE results for the publicly available datasets are compared to those of the classical methods (*SpaMa*, *SpaMaPlus*, and *Schaeck2017*) as well as the average and ensemble CNN models presented in [18], which employed leave-one-session-out cross-validation.

The results obtained demonstrate that non-machine learning solutions can serve as a viable alternative for heart rate estimation. Specifically, our proposed method reduced the MAE for the *PPG-DaLiA* dataset by 1.45 bpm, as shown in Table 2. While the CNN ensemble previously achieved the lowest MAE of 7.65 ± 4.2 bpm, our method obtained an MAE of 6.2 ± 2.0 bpm. Likewise, the MAEs for the *WESAD* dataset showed less than 1 bpm difference between the CNN ensemble and our proposed method, where MAEs of 7.47 ± 3.3 bpm and 8.1 ± 2.6 bpm were obtained, respectively. For the *WESAD* dataset, our method outperformed classical approaches (*SpaMa*, *SpaMaPlus*, and *Schaeck2017*) as well as the CNN average by achieving lower MAE values, as detailed in Table 3. For the *IEEE_Test* dataset, our method also demonstrated a lower MAE (10.8 ± 9.6 bpm) compared to all the other methods, except for *SpaMa* (9.2 ± 11.4 bpm).

However, our algorithm did not perform equally well across all the datasets. Namely, on the *IEEE_Training* dataset, our method yielded an MAE of 6.5 ± 3.6 bpm, which was higher than that of all the other evaluated methods except *SpaMa*. For instance, the CNN ensemble achieved a lower MAE of 4 ± 5.4 bpm. Detailed results for the *IEEE_Training* and *IEEE_Test* datasets are shown in Table 4 and Table 5, respectively.

One objective of collecting the *UTOKYO* dataset was to explore the use of finger PPG and accelerometer signals, as all the other datasets were recorded using wrist-worn devices. A major challenge when analyzing signals from a smart ring is the high level of motion artifacts. These elevated noise levels result from the smart ring being prone to rotating and shifting around the finger during movement, unlike wrist-worn devices, such as smart watches, which usually maintain a fixed orientation. Additionally, 35 running sessions from the dataset were performed outdoors, which exhibited greater motion artifacts than those obtained indoors under laboratory conditions, potentially due to increased exposure to ambient light and greater variability in movements.

To quantify the influence of motion artifacts on the PPG signals of each dataset, we calculated three signal quality indices (SQIs) previously defined by Song et al. [28]. The P index indicates the presence of high-frequency noise by measuring the reduction in local extrema after smoothing. The Q index reflects the influence of baseline wander, and the R index assesses motion artifact contamination based on the variability in the available peak and valley points. Higher values across all three indices indicate better signal quality. To facilitate a comparison between datasets, we also report the relative signal quality index (rSQI), which expresses the signal quality of each dataset relative to the *UTOKYO* dataset. The SQIs are presented in Table 6. All the rSQI values are positive, indicating that the *UTOKYO* dataset contains the noisiest PPG signals among all the datasets evaluated.

The three classical methods and the current method were evaluated on the *UTOKYO* dataset. For the classical methods, the results were obtained with session-specific tuning. Previous findings show that these methods are highly sensitive to parameter setting [18], which motivated the use of session-specific tuning to allow for a comparison between the lowest achievable MAEs and those of our method, which maintained fixed and unchanged parameters. The adjusted parameters for the *SpaMa* methods included the number of PPG and acceleration peaks considered in the spectral analysis, as well as the minimum frequency difference required to remove overlapping peaks [18]. The adjusted parameters for the *Schaeck2017* algorithm included the maximum allowable difference between two consecutive heart rates, the standard deviation used in the Gaussian band stop filter, and the size of the correlation window [29].

The proposed method evaluated on the *UTOKYO* dataset achieved an overall MAE of 7.9 ± 8.2 bpm, whereas the *SpaMa* and *SpaMaPlus* methods yielded an MAE of 37.6 ± 26.2 bpm and 32.3 ± 26.0 bpm, respectively. The *Schaeck2017* algorithm obtained an MAE of 14.1 ± 15.7 bpm. Results are summarized in Table 7.

**Figure 6:**
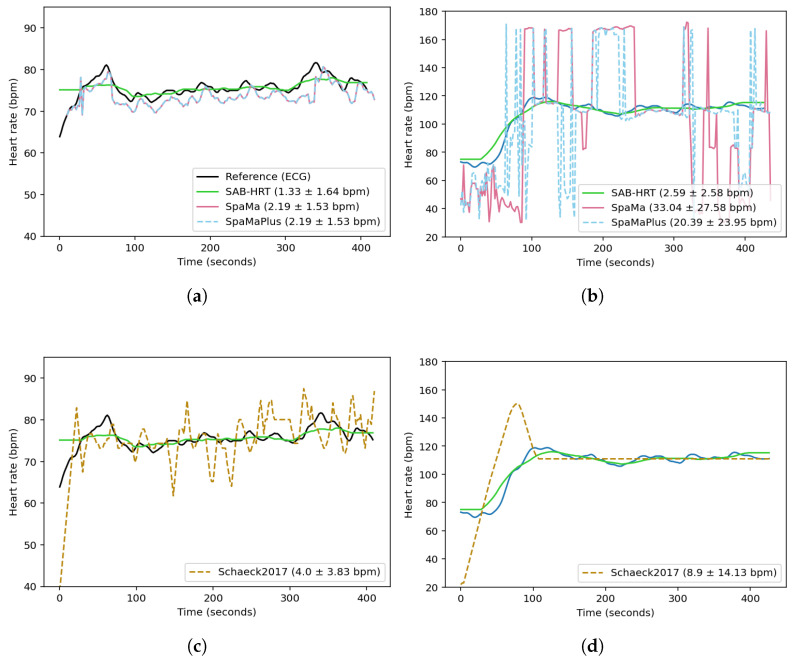
Comparison of the reconstructed heart rates obtained using the three classical methods and this paper’s proposed algorithm across two sessions from the *UTOKYO* dataset. Panels (**a**,**c**) show the results for a walking session (low motion artifacts). Panels (**b**,**d**) show the results for an outdoor running session (high motion artifacts). The legend labels report the method name followed by the MAE ± standard deviation (in bpm), calculated over time for these sessions.

Figure 6 compares the reconstructed heart rates for two sessions from the *UTOKYO* dataset using the three classical methods and the current method. Figure 6a,c correspond to an indoor walking session recorded under low-noise conditions. The *SpaMa* and *SpaMaPlus* methods both yielded MAEs of 2.19 bpm, while the proposed algorithm achieved an MAE of 1.33 bpm, and the*Schaeck2017* method obtained an MAE of 4.0 bpm. In contrast, Figure 6b,d present an outdoor running session with substantially higher noise levels. Under these conditions, the performance of *SpaMa* and *SpaMaPlus* deteriorates significantly, with MAEs of 33.0 and 20.4 bpm, respectively. The current method remains more robust in this high-noise environment, achieving an MAE of 2.6 bpm. The *Schaeck2017* method yields an MAE of 8.9 bpm. These results suggest that while *SpaMa* and *SpaMaPlus* can be effective in evaluating signals with less motion artifacts, their application may be limited in real-world scenarios.

Although the performance of deep learning models is compared to our proposed method for the publicly available datasets, a comparison was not conducted for the *UTOKYO* dataset. This decision was based on the complexity involved in implementing and validating deep learning models within the scope of this work. Thus, further investigation into the application of deep learning methods for finger-based PPG and accelerometer data is warranted.

A common issue with many tracking algorithms is the accumulation of errors over time. Our method tries to mitigate this issue by not relying solely on the previous heart rate value but instead considering multiple values. As such, even in cases where the algorithm makes an incorrect prediction, the error propagation can be limited. However, this improvement comes at the cost of requiring a longer time window, which may be a limitation for real-time applications that demand immediate feedback.

In terms of computational efficiency, our method performed comparably to the three classical approaches. Specifically, across all five datasets, our method was on average 28% faster, albeit using 6% more memory than the *Schaeck2017* method, which was specifically designed for embedded applications and reported to be up to 80 times faster than the JOSS algorithm [29]. By contrast, our method was approximately 2% slower than *SpaMa* and 8% slower than *SpaMaPlus*, while requiring 52% and 49% less memory, respectively. Regarding deep learning-based methods, the CNN architecture presented in [18] utilizes 8.5 million parameters and requires 69.5 million computations per heart rate estimation, making it unsuitable for deployment on resource-constrained devices. However, the authors also introduce a resource-optimized CNN model with only 26 K parameters, designed to operate within a 32 KB memory footprint, which increases the MAE of the *PPG-DaLiA* and *WESAD* datasets to 9.99 ± 5.9 bpm and 8.2 ± 3.6 bpm, respectively. As noted previously, we did not implement this CNN model in the current study, and further investigation is warranted for a complete assessment of its computational efficiency.

Furthermore, the final post-processing step of our algorithm includes a moving average filter. We chose to incorporate this step because commercial PPG- and ECG-based wearables commonly apply similar post-processing to enhance the readability of the displayed heart rate signal for users. However, depending on the target application, particularly those requiring a faster response time, it may be desirable to reduce the filter’s window size. Thus, to evaluate the influence of the window size, we repeated the analysis using a shorter 10-second window. The resulting changes in the MAE were minimal, with variations remaining within ±1.3 bpm across all the datasets. Specifically, the MAE increased for *PPG-DaLiA* (+0.5 bpm), *UTOKYO* (+0.6 bpm), and *WESAD* (+1.2 bpm), while it decreased for *IEEE_Test* (–0.4 bpm) and *IEEE_Training* (–1.3 bpm).

An additional drawback of the current method is its reliance on the presence of detectable PSD peaks. In scenarios where the input signal is entirely corrupted or absent, such as when the sensor loses consistent contact with the skin, this method may be less reliable than machine learning or deep learning models that can leverage other data sources and health trends to generate HR estimates.

## 4. Conclusions

Our approach demonstrates that non-machine learning methods can deliver performance comparable to more complex deep learning models, offering a viable alternative for heart rate estimation. This method is especially valuable in contexts where deep learning may not be suitable, such as when computational resources are limited, annotated datasets are scarce, or simpler, more interpretable solutions are preferred. By leveraging signal processing and algorithmic solutions, our method expands the toolkit for wearable technology, providing an efficient and lightweight solution.

Moreover, many existing algorithms depend on subject-specific or dataset-specific tuning, which can restrict their generalization in real-world applications where ground truth data is unavailable for calibration. To address this challenge, our method proposes a one-size-fits-all approach, with parameters not tailored to any specific dataset, yet still achieving results comparable to other algorithms. However, in cases where calibration data is available, additional tuning of the parameters can further improve performance.

## Figures and Tables

**Table 1:** Mean and standard deviation of ECG-derived heart rates in beats per minute (bpm)across datasets.

Dataset	Mean HR (bpm)	Conditions
PPG-DaLiA	89.43 ± 22.83	Wrist-based
WESAD	76.51 ± 13.03	
IEEE_Training	135.95 ± 24.30	
IEEE_Test	115.39 ± 31.08	
UTOKYO	124.04 ± 30.96	Finger-based
Warm-up	136.92 ± 23.7	
Walking	78.55 ± 6.94	
Indoor running	139.22 ± 20.73	
Outdoor running	130.58 ± 26.21	

**Table 2:** Evaluation results on the dataset *PPG-DaLiA* achieved with the three classical methods (leave-one-session-out cross-validation), the best performing CNN architecture (ensemble prediction; results averaging 7 repetitions), and this paper’s proposed algorithm. The results are given in MAE (bpm) across all the subjects.

Method	MAE [bpm]	Reference
SpaMa	15.6±7.5	
SpaMaPlus	11.06±4.8	[18]
Schaeck2017	20.45±7.1	
CNN average	8.82±3.8	
CNN ensemble	7.65±4.2	
SAB-HRT	6.2±2.0	This work

MAE = mean absolute error; bpm = beats per minute.

**Table 3:** Evaluation results on the dataset *WESAD* achieved with the three classical methods (leave-one-session-out cross-validation), the best performing CNN architecture (ensemble prediction; results averaging 7 repetitions), and this paper’s proposed algorithm. The results are given in MAE (bpm) across all the subjects.

Method	MAE [bpm]	Reference
SpaMa	11.51±3.7	
SpaMaPlus	9.45±2.9	[18]
Schaeck2017	19.97±8.1	
CNN average	8.42±3	
CNN ensemble	7.47±3.3	
SAB-HRT	8.1±2.6	This work

**Table 4:** Evaluation results on the dataset *IEEE_Training* achieved with the three classical methods (leave-one-session-out cross-validation), the best performing CNN architecture (ensemble predictions), and this paper’s proposed algorithm. The results are given in MAE (bpm) across all the subjects.

Method	MAE [bpm]	Reference
SpaMa	13.1±20.7	
SpaMaPlus	4.25±5.9	[18]
Schaeck2017	2.91±4.6	
CNN ensemble	4±5.4	
SAB-HRT	6.5±3.6	This work

**Table 5:** Evaluation results on the dataset *IEEE_Test* achieved with the three classical methods (leave-one-session-out cross-validation), the best performing CNN architecture (ensemble predictions), and this paper’s proposed algorithm. The results are given in MAE (bpm) across all the subjects.

Method	MAE [bpm]	Reference
SpaMa	9.2±11.4	
SpaMaPlus	12.31±15.5	[18]
Schaeck2017	24.65±24	
CNN ensemble	16.51±16.1	
SAB-HRT	10.8±9.6	This work

**Table 6:** Average PPG signal quality for each dataset using the P, Q, and R indices and the relative signal quality (rSQI) with respect to the *UTOKYO* dataset.

Dataset *i*	Dataset	P	Q	R	${\mathrm{rSQI}}_{i5}$
1	PPG-DaLiA	0.87	0.53	0.38	2.16
2	WESAD	0.86	0.54	0.25	1.82
3	IEEE_Training	0.56	0.70	0.48	1.42
4	IEEE_Test	0.23	0.67	0.45	0.008
5	UTOKYO	0.25	0.64	0.42	

**Table 7:** Evaluation results on the dataset *UTOKYO* achieved with the three classical methods (session-optimized) and this paper’s proposed algorithm. The results are given in MAE (bpm) across all the subjects.

Method	MAE [bpm]
SpaMa	37.6±26.2
SpaMaPlus	32.3±26.0
Schaeck2017	14.1±15.7
SAB-HRT	7.9±8.2

## Data Availability

The data presented in this study are available on request from the corresponding author due to institutional and commercial restrictions. Access can be granted for non-commercial, academic research purposes only and is subject to approval by SOXAI Inc. and The University of Tokyo.
